# Cyclo­hexyl­ammonium acetate–*N*,*N*′,*N*′′-tricyclo­hexyl­phospho­ric triamide (1/1)

**DOI:** 10.1107/S1600536812028589

**Published:** 2012-06-30

**Authors:** Mehrdad Pourayoubi, Mojtaba Keikha, Arnold L. Rheingold, James A. Golen

**Affiliations:** a Department of Chemistry, Ferdowsi University of Mashhad, Mashhad, Iran; b Department of Chemistry, University of California, San Diego, 9500 Gilman Drive, La Jolla, CA 92093, USA

## Abstract

In the phospho­ric triamide mol­ecule of the title compound, C_6_H_14_N^+^·C_2_H_3_O_2_^−^·C_18_H_36_N_3_OP, the P atom displays a distorted tetra­hedral geometry and the cyclo­hexyl rings adopt chair conformations with the NH groups in equatorial positions. In the crystal, the cations, anions and phosphoric triamide mol­ecules are linked *via* N—H⋯O hydrogen bonds into a two-dimensional array parallel to the *bc* plane. The O atom of the P(O) group acts as a double-hydrogen-bond acceptor.

## Related literature

For background to phospho­ric triamide mol­ecules and for bond lengths and angles in related structures, see: Pourayoubi, Tarahhomi *et al.* (2012 ▶); Sabbaghi *et al.* (2011 ▶). For a definition of double-hydrogen-bond acceptor, see: Pourayoubi, Nečas & Negari (2012 ▶). For hydrolysis of compounds containing a C N bond, see: Vollhardt & Schore (1998 ▶).
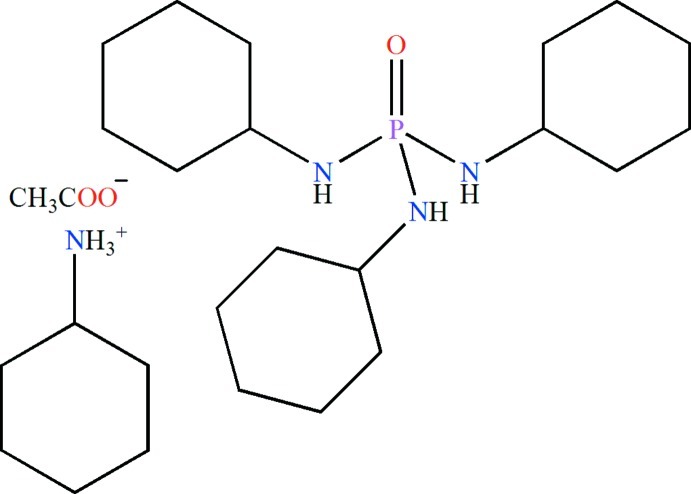


## Experimental

### 

#### Crystal data

C_6_H_14_N^+^·C_2_H_3_O_2_^−^·C_18_H_36_N_3_OP
*M* *_r_* = 500.69
Monoclinic, 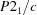

*a* = 12.7663 (8) Å
*b* = 10.9011 (7) Å
*c* = 21.2791 (13) Å
β = 104.523 (3)°
*V* = 2866.7 (3) Å^3^
*Z* = 4
Mo *K*α radiation
μ = 0.13 mm^−1^
*T* = 90 K
0.35 × 0.25 × 0.20 mm

#### Data collection

Bruker APEXII CCD diffractometer
Absorption correction: multi-scan (*SADABS*; Sheldrick, 2004 ▶) *T*_min_ = 0.957, *T*_max_ = 0.975
21979 measured reflections
5898 independent reflections
4702 reflections with *I* > 2σ(*I*)
*R*_int_ = 0.043

#### Refinement

*R*[*F* ^2^ > 2σ(*F* ^2^)] = 0.044
*wR*(*F* ^2^) = 0.119
*S* = 0.97
5898 reflections
326 parameters
6 restraints
H atoms treated by a mixture of independent and constrained refinement
Δρ_max_ = 0.47 e Å^−3^
Δρ_min_ = −0.47 e Å^−3^

### 

Data collection: *APEX2* (Bruker, 2005 ▶); cell refinement: *SAINT* (Bruker, 2005 ▶); data reduction: *SAINT*; program(s) used to solve structure: *SHELXS97* (Sheldrick, 2008 ▶); program(s) used to refine structure: *SHELXL97* (Sheldrick, 2008 ▶); molecular graphics: *SHELXTL* (Sheldrick, 2008 ▶) and *Mercury* (Macrae *et al.*, 2008 ▶); software used to prepare material for publication: *SHELXTL* and *enCIFer* (Allen *et al.*, 2004 ▶).

## Supplementary Material

Crystal structure: contains datablock(s) I, global. DOI: 10.1107/S1600536812028589/ff2071sup1.cif

Structure factors: contains datablock(s) I. DOI: 10.1107/S1600536812028589/ff2071Isup2.hkl

Additional supplementary materials:  crystallographic information; 3D view; checkCIF report

## Figures and Tables

**Table 1:** Hydrogen-bond geometry (Å, °)

*D*—H⋯*A*	*D*—H	H⋯*A*	*D*⋯*A*	*D*—H⋯*A*
N2—H2*N*⋯O1^i^	0.87 (1)	2.14 (2)	3.0049 (18)	171 (2)
N3—H3*N*⋯O2^ii^	0.84 (1)	2.05 (2)	2.8837 (18)	173 (2)
N1—H1*N*⋯O3	0.86 (1)	2.21 (2)	3.0394 (18)	163 (2)
N4—H4*NC*⋯O1^iii^	0.89 (1)	2.05 (2)	2.9445 (18)	178 (2)
N4—H4*NB*⋯O3^iv^	0.89 (2)	1.94 (2)	2.7666 (19)	155 (2)
N4—H4*NA*⋯O2^v^	0.88 (2)	1.83 (2)	2.6992 (19)	169 (2)

Symmetry codes: (i) 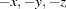; (ii) 
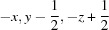
; (iii) 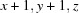; (iv) 
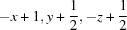
; (v) 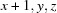.

**Fig. 1.**
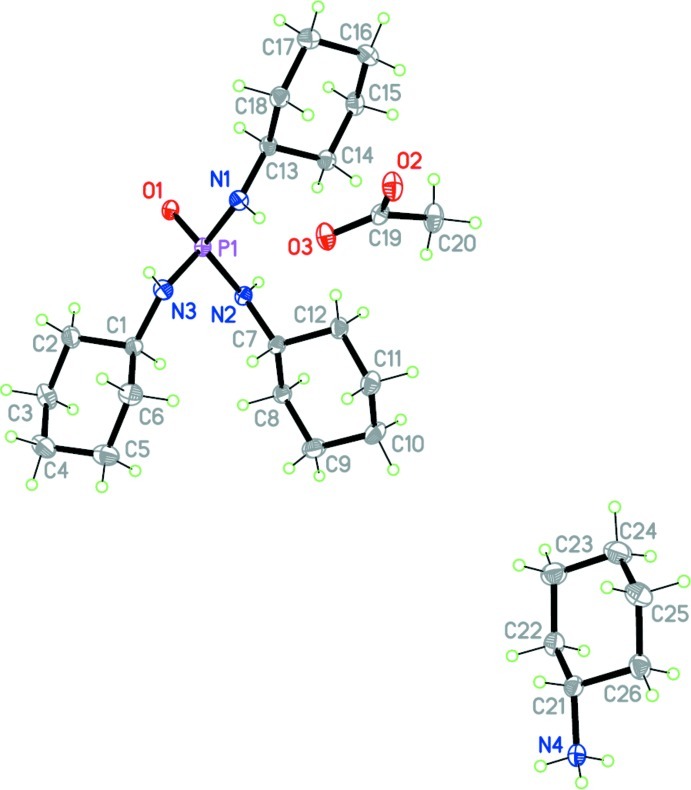
An ORTEP-style plot and atom labeling scheme for the title cocrystal. Displacement ellipsoids are given at 50% probability level and H atoms are drawn as small spheres of arbitrary radii.

**Fig. 2.**
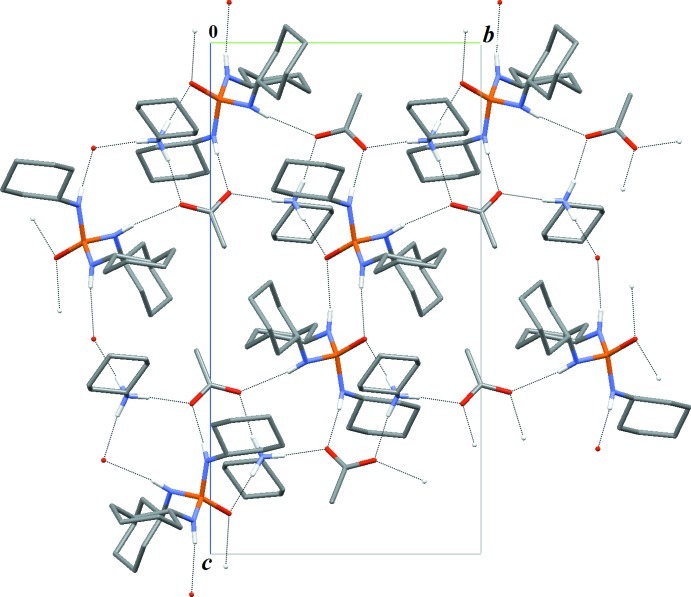
A view of the two-dimensional arrangement with the N—H···O hydrogen bonds parallel to bc plane. The N–H···O hydrogen bonds are shown as dashed lines and the H atoms bound to C atoms have been omitted for clarity.

## supplementary crystallographic information

### Comment

The X-ray determination of the title co-crystal, P(O)(NHC_6_H_11_)_3_,(C_6_H_11_NH_3_^+^)(CH_3_COO^-^), (Fig. 1) was performed following to our previous study on synthesis and crystal structure determination of phosphoric triamide compounds (Pourayoubi, Tarahhomi *et al.*, 2012 and Sabbaghi *et al.*, 2011).

The cyclohexyl rings of phosphoric triamide molecule and also in the C_6_H_11_NH_3_^+^ cation have the chair conformation and the NH and NH_3_^+^ groups are in the equatorial position of the rings. In P(O)(NHC_6_H_11_)_3_, the P atom exists in a distorted tetrahedral environment with the P—N bond lengths of 1.6315 (14) Å, 1.6440 (14) Å and 1.6463 (14) Å (for P1—N1). The P═O bond length and the P—N—C bond angles are standard for the phosphoric triamides (Pourayoubi, Tarahhomi *et al.*, 2012 and Sabbaghi *et al.*, 2011).

In the crystal, the oxygen atom of the P═O group acts as a double-hydrogen bond acceptor (Pourayoubi, Nečas & Negari, 2012), forming the P═O···[H—N][H—N] grouping with one N—H unit of a neighboring P(O)(NHC_6_H_11_)_3_ molecule and one N—H unit of C_6_H_11_NH_3_^+^ cation. Other N—H units of P(O)(NHC_6_H_11_)_3_ and N—H units of C_6_H_11_NH_3_^+^ are involved in the N—H···O hydrogen bonds with the O atoms of acetate anion. These N—H···O hydrogen bonds form a two-dimensional arrangement parallel to *bc* plane (Fig. 2).

### Experimental

The title co-crystal was obtained fortuitously from a reaction between phosphoryl chloride and cyclohexylamine in acetonitrile at 273 K (4 h) and then the treatment of dibenzylamine at ice bath temperature. The presence of acetate anion is attributed to the hydrolysis of acetonitrile in acidic media of reaction (Vollhardt & Schore, 1998).

### Refinement

Structure was solved by direct methods and all non-hydrogen atoms were refined by full matrix least squares on F^2^. All nitrogen hydrogen atoms were found from a Fourier difference map and were refined isotropically with N—H distance of 0.87 (2) Å and 1.2*U*_eq_ of parent N atom. All other H atoms were placed in calculated positions and treated as riding on their parent atoms with C—H = 0.980 Å (CH_3_), 0.990 Å (CH_2_), and 1.000 Å (CH) with 1.5*U*_eq_ for methyl groups and 1.2*U*_eq_ for other H atoms.

### Crystal data

**Table:**

C_6_H_14_N^+^·C_2_H_3_O_2_^−^·C_18_H_36_N_3_OP	*F*(000) = 1104
*M**_r_* = 500.69	*D*_x_ = 1.160 Mg m^−^^3^
Monoclinic, *P*2_1_/*c*	Mo *K*α radiation, λ = 0.71073 Å
Hall symbol: -P 2ybc	Cell parameters from 4565 reflections
*a* = 12.7663 (8) Å	θ = 2.5–26.4°
*b* = 10.9011 (7) Å	µ = 0.13 mm^−^^1^
*c* = 21.2791 (13) Å	*T* = 90 K
β = 104.523 (3)°	Block, colourless
*V* = 2866.7 (3) Å^3^	0.35 × 0.25 × 0.20 mm
*Z* = 4	

### Data collection

**Table:**

Bruker APEXII CCD diffractometer	5898 independent reflections
Radiation source: fine-focus sealed tube	4702 reflections with *I* > 2σ(*I*)
Graphite monochromator	*R*_int_ = 0.043
φ and ω scans	θ_max_ = 26.5°, θ_min_ = 1.7°
Absorption correction: multi-scan (*SADABS*; Sheldrick, 2004)	*h* = −13→16
*T*_min_ = 0.957, *T*_max_ = 0.975	*k* = −12→13
21979 measured reflections	*l* = −25→26

### Refinement

**Table:**

Refinement on *F*^2^	Primary atom site location: structure-invariant direct methods
Least-squares matrix: full	Secondary atom site location: difference Fourier map
*R*[*F*^2^ > 2σ(*F*^2^)] = 0.044	Hydrogen site location: inferred from neighbouring sites
*wR*(*F*^2^) = 0.119	H atoms treated by a mixture of independent and constrained refinement
*S* = 0.97	*w* = 1/[σ^2^(*F*_o_^2^) + (0.0636*P*)^2^ + 1.5804*P*] where *P* = (*F*_o_^2^ + 2*F*_c_^2^)/3
5898 reflections	(Δ/σ)_max_ < 0.001
326 parameters	Δρ_max_ = 0.47 e Å^−^^3^
6 restraints	Δρ_min_ = −0.47 e Å^−^^3^

### Special details

**Table:**

Geometry. All e.s.d.'s (except the e.s.d. in the dihedral angle between two l.s. planes) are estimated using the full covariance matrix. The cell e.s.d.'s are taken into account individually in the estimation of e.s.d.'s in distances, angles and torsion angles; correlations between e.s.d.'s in cell parameters are only used when they are defined by crystal symmetry. An approximate (isotropic) treatment of cell e.s.d.'s is used for estimating e.s.d.'s involving l.s. planes.
Refinement. Refinement of *F*^2^ against ALL reflections. The weighted *R*-factor *wR* and goodness of fit *S* are based on *F*^2^, conventional *R*-factors *R* are based on *F*, with *F* set to zero for negative *F*^2^. The threshold expression of *F*^2^ > σ(*F*^2^) is used only for calculating *R*-factors(gt) *etc*. and is not relevant to the choice of reflections for refinement. *R*-factors based on *F*^2^ are statistically about twice as large as those based on *F*, and *R*- factors based on ALL data will be even larger.

### Fractional atomic coordinates and isotropic or equivalent isotropic displacement parameters (Å^2^)

**Table:**

	*x*	*y*	*z*	*U*_iso_*/*U*_eq_	
P1	0.03749 (3)	0.03310 (4)	0.110490 (19)	0.01413 (12)	
O1	−0.03516 (9)	−0.06943 (10)	0.07941 (5)	0.0162 (3)	
O2	−0.03699 (11)	0.56848 (11)	0.20581 (6)	0.0235 (3)	
O3	0.03597 (10)	0.39451 (11)	0.18349 (6)	0.0226 (3)	
N1	−0.03942 (11)	0.14670 (13)	0.12349 (6)	0.0162 (3)	
H1N	−0.0051 (14)	0.2092 (15)	0.1427 (9)	0.019*	
N2	0.12192 (11)	0.06325 (12)	0.06552 (7)	0.0153 (3)	
H2N	0.0942 (14)	0.0571 (17)	0.0240 (7)	0.018*	
N3	0.11772 (11)	0.01218 (13)	0.18250 (7)	0.0169 (3)	
H3N	0.0902 (15)	0.0241 (18)	0.2140 (8)	0.020*	
N4	0.90968 (12)	0.80813 (14)	0.19022 (7)	0.0175 (3)	
H4NC	0.9261 (15)	0.8438 (17)	0.1561 (8)	0.021*	
H4NB	0.9447 (14)	0.8426 (17)	0.2274 (8)	0.021*	
H4NA	0.9338 (15)	0.7323 (14)	0.1928 (9)	0.021*	
C1	0.21703 (13)	−0.06129 (15)	0.19470 (8)	0.0165 (3)	
H1A	0.2472	−0.0528	0.1558	0.020*	
C2	0.19909 (15)	−0.19790 (16)	0.20397 (9)	0.0223 (4)	
H2B	0.1455	−0.2303	0.1656	0.027*	
H2A	0.1701	−0.2100	0.2425	0.027*	
C3	0.30587 (16)	−0.26732 (17)	0.21303 (9)	0.0277 (4)	
H3B	0.2940	−0.3549	0.2216	0.033*	
H3A	0.3301	−0.2625	0.1724	0.033*	
C4	0.39406 (16)	−0.21611 (18)	0.26870 (9)	0.0279 (4)	
H4A	0.3752	−0.2326	0.3103	0.033*	
H4B	0.4632	−0.2582	0.2699	0.033*	
C5	0.40827 (15)	−0.07895 (19)	0.26147 (9)	0.0281 (4)	
H5A	0.4379	−0.0634	0.2235	0.034*	
H5B	0.4608	−0.0473	0.3005	0.034*	
C6	0.30118 (14)	−0.01101 (17)	0.25267 (9)	0.0237 (4)	
H6A	0.2747	−0.0201	0.2924	0.028*	
H6B	0.3124	0.0775	0.2461	0.028*	
C7	0.21298 (13)	0.14884 (15)	0.08461 (8)	0.0156 (3)	
H7A	0.2431	0.1405	0.1325	0.019*	
C8	0.30137 (13)	0.11132 (16)	0.05214 (8)	0.0192 (4)	
H8A	0.2731	0.1162	0.0044	0.023*	
H8B	0.3223	0.0251	0.0635	0.023*	
C9	0.40097 (15)	0.19339 (17)	0.07308 (10)	0.0262 (4)	
H9A	0.4355	0.1792	0.1196	0.031*	
H9B	0.4538	0.1712	0.0480	0.031*	
C10	0.37214 (15)	0.32891 (17)	0.06253 (9)	0.0251 (4)	
H10A	0.4371	0.3793	0.0810	0.030*	
H10B	0.3485	0.3460	0.0154	0.030*	
C11	0.28244 (15)	0.36423 (16)	0.09441 (9)	0.0244 (4)	
H11A	0.2623	0.4511	0.0845	0.029*	
H11B	0.3090	0.3560	0.1421	0.029*	
C12	0.18287 (14)	0.28355 (15)	0.07060 (9)	0.0208 (4)	
H12A	0.1533	0.2954	0.0233	0.025*	
H12B	0.1264	0.3074	0.0928	0.025*	
C13	−0.13969 (13)	0.17719 (15)	0.07496 (8)	0.0165 (3)	
H13A	−0.1773	0.0984	0.0591	0.020*	
C14	−0.12088 (14)	0.24561 (16)	0.01597 (8)	0.0181 (4)	
H14A	−0.0792	0.3214	0.0306	0.022*	
H14B	−0.0775	0.1935	−0.0061	0.022*	
C15	−0.22712 (15)	0.27884 (16)	−0.03186 (8)	0.0226 (4)	
H15A	−0.2644	0.2028	−0.0508	0.027*	
H15B	−0.2119	0.3280	−0.0677	0.027*	
C16	−0.30106 (15)	0.35149 (18)	0.00041 (9)	0.0256 (4)	
H16A	−0.3711	0.3664	−0.0313	0.031*	
H16B	−0.2677	0.4320	0.0148	0.031*	
C17	−0.32031 (15)	0.28178 (18)	0.05861 (9)	0.0275 (4)	
H17A	−0.3604	0.2052	0.0435	0.033*	
H17B	−0.3651	0.3324	0.0804	0.033*	
C18	−0.21338 (14)	0.25052 (16)	0.10695 (8)	0.0207 (4)	
H18A	−0.1765	0.3273	0.1251	0.025*	
H18B	−0.2281	0.2024	0.1433	0.025*	
C19	0.00103 (13)	0.50061 (15)	0.16902 (8)	0.0170 (3)	
C20	0.00121 (17)	0.55092 (16)	0.10254 (9)	0.0251 (4)	
H20A	0.0599	0.5127	0.0874	0.038*	
H20B	0.0121	0.6399	0.1054	0.038*	
H20C	−0.0682	0.5326	0.0719	0.038*	
C21	0.79125 (14)	0.81068 (15)	0.18393 (8)	0.0184 (4)	
H21A	0.7770	0.7760	0.2245	0.022*	
C22	0.73229 (14)	0.73242 (16)	0.12695 (8)	0.0224 (4)	
H22A	0.7564	0.6462	0.1344	0.027*	
H22B	0.7505	0.7613	0.0869	0.027*	
C23	0.61009 (15)	0.73912 (18)	0.11812 (9)	0.0284 (4)	
H23A	0.5911	0.6999	0.1558	0.034*	
H23B	0.5736	0.6929	0.0787	0.034*	
C24	0.56931 (16)	0.87123 (19)	0.11199 (10)	0.0323 (5)	
H24A	0.5791	0.9072	0.0711	0.039*	
H24B	0.4911	0.8723	0.1102	0.039*	
C25	0.63031 (15)	0.94796 (19)	0.16928 (10)	0.0302 (4)	
H25A	0.6056	1.0342	0.1629	0.036*	
H25B	0.6137	0.9174	0.2095	0.036*	
C26	0.75158 (15)	0.94279 (16)	0.17677 (9)	0.0242 (4)	
H26A	0.7890	0.9905	0.2155	0.029*	
H26B	0.7691	0.9802	0.1383	0.029*	

### Atomic displacement parameters (Å^2^)

**Table:**

	*U*^11^	*U*^22^	*U*^33^	*U*^12^	*U*^13^	*U*^23^
P1	0.0168 (2)	0.0128 (2)	0.0132 (2)	0.00017 (16)	0.00465 (16)	0.00000 (15)
O1	0.0194 (6)	0.0131 (6)	0.0164 (6)	−0.0014 (4)	0.0051 (5)	−0.0006 (4)
O2	0.0365 (8)	0.0165 (6)	0.0221 (6)	0.0046 (5)	0.0157 (6)	0.0018 (5)
O3	0.0317 (7)	0.0137 (6)	0.0225 (6)	0.0031 (5)	0.0068 (5)	0.0004 (5)
N1	0.0182 (7)	0.0144 (7)	0.0153 (7)	−0.0001 (6)	0.0030 (6)	−0.0026 (5)
N2	0.0176 (7)	0.0156 (7)	0.0131 (7)	−0.0019 (5)	0.0043 (6)	−0.0006 (5)
N3	0.0193 (7)	0.0189 (7)	0.0140 (7)	0.0030 (6)	0.0068 (6)	0.0000 (6)
N4	0.0215 (8)	0.0137 (7)	0.0184 (7)	0.0004 (6)	0.0071 (6)	0.0008 (6)
C1	0.0167 (8)	0.0169 (8)	0.0168 (8)	0.0018 (6)	0.0060 (7)	0.0011 (6)
C2	0.0246 (9)	0.0192 (9)	0.0220 (9)	0.0002 (7)	0.0036 (7)	0.0040 (7)
C3	0.0324 (11)	0.0225 (10)	0.0272 (10)	0.0075 (8)	0.0056 (8)	0.0055 (8)
C4	0.0250 (10)	0.0378 (11)	0.0216 (9)	0.0108 (8)	0.0073 (8)	0.0062 (8)
C5	0.0204 (9)	0.0377 (11)	0.0245 (10)	0.0018 (8)	0.0025 (8)	−0.0001 (8)
C6	0.0234 (9)	0.0258 (10)	0.0205 (9)	−0.0006 (8)	0.0032 (7)	−0.0031 (7)
C7	0.0168 (8)	0.0140 (8)	0.0159 (8)	−0.0015 (6)	0.0039 (6)	−0.0010 (6)
C8	0.0187 (9)	0.0157 (8)	0.0238 (9)	0.0004 (7)	0.0064 (7)	−0.0004 (7)
C9	0.0189 (9)	0.0248 (10)	0.0355 (11)	−0.0012 (7)	0.0081 (8)	−0.0027 (8)
C10	0.0242 (10)	0.0206 (9)	0.0317 (10)	−0.0073 (7)	0.0095 (8)	−0.0035 (8)
C11	0.0302 (10)	0.0162 (9)	0.0285 (10)	−0.0021 (7)	0.0107 (8)	−0.0015 (7)
C12	0.0233 (9)	0.0147 (8)	0.0266 (9)	0.0015 (7)	0.0106 (8)	0.0019 (7)
C13	0.0180 (8)	0.0137 (8)	0.0179 (8)	0.0004 (6)	0.0046 (7)	−0.0001 (6)
C14	0.0205 (9)	0.0163 (8)	0.0183 (8)	0.0003 (7)	0.0061 (7)	−0.0006 (7)
C15	0.0273 (10)	0.0194 (9)	0.0198 (9)	−0.0017 (7)	0.0033 (7)	0.0028 (7)
C16	0.0213 (9)	0.0260 (10)	0.0284 (10)	0.0053 (7)	0.0040 (8)	0.0063 (8)
C17	0.0209 (10)	0.0301 (11)	0.0335 (10)	0.0042 (8)	0.0106 (8)	0.0064 (8)
C18	0.0224 (9)	0.0189 (9)	0.0231 (9)	0.0012 (7)	0.0103 (7)	0.0021 (7)
C19	0.0187 (8)	0.0143 (8)	0.0182 (8)	−0.0031 (6)	0.0050 (7)	−0.0007 (6)
C20	0.0401 (11)	0.0169 (9)	0.0217 (9)	0.0004 (8)	0.0141 (8)	0.0000 (7)
C21	0.0196 (9)	0.0168 (8)	0.0198 (8)	−0.0005 (7)	0.0069 (7)	0.0013 (6)
C22	0.0264 (10)	0.0196 (9)	0.0209 (9)	−0.0018 (7)	0.0056 (7)	0.0009 (7)
C23	0.0238 (10)	0.0326 (11)	0.0269 (10)	−0.0040 (8)	0.0031 (8)	0.0006 (8)
C24	0.0228 (10)	0.0390 (12)	0.0334 (11)	0.0046 (8)	0.0041 (8)	0.0048 (9)
C25	0.0251 (10)	0.0309 (11)	0.0349 (11)	0.0075 (8)	0.0080 (8)	0.0002 (8)
C26	0.0268 (10)	0.0185 (9)	0.0280 (10)	0.0029 (7)	0.0079 (8)	−0.0001 (7)

### Geometric parameters (Å, º)

**Table:**

P1—O1	1.4964 (12)	C10—H10B	0.9900
P1—N3	1.6315 (14)	C11—C12	1.524 (2)
P1—N2	1.6440 (14)	C11—H11A	0.9900
P1—N1	1.6463 (14)	C11—H11B	0.9900
O2—C19	1.260 (2)	C12—H12A	0.9900
O3—C19	1.250 (2)	C12—H12B	0.9900
N1—C13	1.467 (2)	C13—C18	1.519 (2)
N1—H1N	0.857 (14)	C13—C14	1.530 (2)
N2—C7	1.466 (2)	C13—H13A	1.0000
N2—H2N	0.868 (14)	C14—C15	1.521 (2)
N3—C1	1.467 (2)	C14—H14A	0.9900
N3—H3N	0.841 (14)	C14—H14B	0.9900
N4—C21	1.484 (2)	C15—C16	1.521 (3)
N4—H4NC	0.894 (14)	C15—H15A	0.9900
N4—H4NB	0.888 (15)	C15—H15B	0.9900
N4—H4NA	0.879 (15)	C16—C17	1.525 (3)
C1—C6	1.520 (2)	C16—H16A	0.9900
C1—C2	1.527 (2)	C16—H16B	0.9900
C1—H1A	1.0000	C17—C18	1.527 (3)
C2—C3	1.529 (3)	C17—H17A	0.9900
C2—H2B	0.9900	C17—H17B	0.9900
C2—H2A	0.9900	C18—H18A	0.9900
C3—C4	1.521 (3)	C18—H18B	0.9900
C3—H3B	0.9900	C19—C20	1.518 (2)
C3—H3A	0.9900	C20—H20A	0.9800
C4—C5	1.519 (3)	C20—H20B	0.9800
C4—H4A	0.9900	C20—H20C	0.9800
C4—H4B	0.9900	C21—C22	1.519 (2)
C5—C6	1.525 (3)	C21—C26	1.521 (2)
C5—H5A	0.9900	C21—H21A	1.0000
C5—H5B	0.9900	C22—C23	1.526 (3)
C6—H6A	0.9900	C22—H22A	0.9900
C6—H6B	0.9900	C22—H22B	0.9900
C7—C8	1.519 (2)	C23—C24	1.526 (3)
C7—C12	1.528 (2)	C23—H23A	0.9900
C7—H7A	1.0000	C23—H23B	0.9900
C8—C9	1.527 (2)	C24—C25	1.522 (3)
C8—H8A	0.9900	C24—H24A	0.9900
C8—H8B	0.9900	C24—H24B	0.9900
C9—C10	1.525 (3)	C25—C26	1.517 (3)
C9—H9A	0.9900	C25—H25A	0.9900
C9—H9B	0.9900	C25—H25B	0.9900
C10—C11	1.520 (2)	C26—H26A	0.9900
C10—H10A	0.9900	C26—H26B	0.9900
			
O1—P1—N3	118.96 (7)	C11—C12—C7	109.97 (14)
O1—P1—N2	108.44 (7)	C11—C12—H12A	109.7
N3—P1—N2	103.05 (7)	C7—C12—H12A	109.7
O1—P1—N1	107.84 (7)	C11—C12—H12B	109.7
N3—P1—N1	101.99 (7)	C7—C12—H12B	109.7
N2—P1—N1	116.95 (7)	H12A—C12—H12B	108.2
C13—N1—P1	120.26 (11)	N1—C13—C18	109.44 (13)
C13—N1—H1N	114.0 (13)	N1—C13—C14	113.53 (13)
P1—N1—H1N	115.0 (13)	C18—C13—C14	110.69 (14)
C7—N2—P1	123.78 (11)	N1—C13—H13A	107.7
C7—N2—H2N	115.0 (12)	C18—C13—H13A	107.7
P1—N2—H2N	114.7 (12)	C14—C13—H13A	107.7
C1—N3—P1	123.63 (11)	C15—C14—C13	111.59 (14)
C1—N3—H3N	117.3 (13)	C15—C14—H14A	109.3
P1—N3—H3N	116.0 (13)	C13—C14—H14A	109.3
C21—N4—H4NC	111.1 (12)	C15—C14—H14B	109.3
C21—N4—H4NB	110.3 (12)	C13—C14—H14B	109.3
H4NC—N4—H4NB	111.7 (18)	H14A—C14—H14B	108.0
C21—N4—H4NA	110.9 (13)	C14—C15—C16	111.84 (15)
H4NC—N4—H4NA	108.0 (17)	C14—C15—H15A	109.2
H4NB—N4—H4NA	104.6 (18)	C16—C15—H15A	109.2
N3—C1—C6	110.51 (14)	C14—C15—H15B	109.2
N3—C1—C2	113.85 (14)	C16—C15—H15B	109.2
C6—C1—C2	110.19 (14)	H15A—C15—H15B	107.9
N3—C1—H1A	107.3	C15—C16—C17	110.59 (15)
C6—C1—H1A	107.3	C15—C16—H16A	109.5
C2—C1—H1A	107.3	C17—C16—H16A	109.5
C1—C2—C3	109.95 (15)	C15—C16—H16B	109.5
C1—C2—H2B	109.7	C17—C16—H16B	109.5
C3—C2—H2B	109.7	H16A—C16—H16B	108.1
C1—C2—H2A	109.7	C16—C17—C18	111.06 (15)
C3—C2—H2A	109.7	C16—C17—H17A	109.4
H2B—C2—H2A	108.2	C18—C17—H17A	109.4
C4—C3—C2	112.27 (16)	C16—C17—H17B	109.4
C4—C3—H3B	109.2	C18—C17—H17B	109.4
C2—C3—H3B	109.2	H17A—C17—H17B	108.0
C4—C3—H3A	109.2	C13—C18—C17	111.40 (15)
C2—C3—H3A	109.2	C13—C18—H18A	109.3
H3B—C3—H3A	107.9	C17—C18—H18A	109.3
C5—C4—C3	111.49 (15)	C13—C18—H18B	109.3
C5—C4—H4A	109.3	C17—C18—H18B	109.3
C3—C4—H4A	109.3	H18A—C18—H18B	108.0
C5—C4—H4B	109.3	O3—C19—O2	124.04 (15)
C3—C4—H4B	109.3	O3—C19—C20	118.69 (15)
H4A—C4—H4B	108.0	O2—C19—C20	117.26 (15)
C4—C5—C6	111.38 (16)	C19—C20—H20A	109.5
C4—C5—H5A	109.4	C19—C20—H20B	109.5
C6—C5—H5A	109.4	H20A—C20—H20B	109.5
C4—C5—H5B	109.4	C19—C20—H20C	109.5
C6—C5—H5B	109.4	H20A—C20—H20C	109.5
H5A—C5—H5B	108.0	H20B—C20—H20C	109.5
C1—C6—C5	110.73 (15)	N4—C21—C22	110.56 (14)
C1—C6—H6A	109.5	N4—C21—C26	109.43 (14)
C5—C6—H6A	109.5	C22—C21—C26	111.46 (15)
C1—C6—H6B	109.5	N4—C21—H21A	108.4
C5—C6—H6B	109.5	C22—C21—H21A	108.4
H6A—C6—H6B	108.1	C26—C21—H21A	108.4
N2—C7—C8	109.29 (13)	C21—C22—C23	110.98 (15)
N2—C7—C12	114.46 (14)	C21—C22—H22A	109.4
C8—C7—C12	110.43 (13)	C23—C22—H22A	109.4
N2—C7—H7A	107.5	C21—C22—H22B	109.4
C8—C7—H7A	107.5	C23—C22—H22B	109.4
C12—C7—H7A	107.5	H22A—C22—H22B	108.0
C7—C8—C9	111.62 (14)	C22—C23—C24	111.85 (16)
C7—C8—H8A	109.3	C22—C23—H23A	109.2
C9—C8—H8A	109.3	C24—C23—H23A	109.2
C7—C8—H8B	109.3	C22—C23—H23B	109.2
C9—C8—H8B	109.3	C24—C23—H23B	109.2
H8A—C8—H8B	108.0	H23A—C23—H23B	107.9
C10—C9—C8	111.84 (15)	C25—C24—C23	110.81 (16)
C10—C9—H9A	109.2	C25—C24—H24A	109.5
C8—C9—H9A	109.2	C23—C24—H24A	109.5
C10—C9—H9B	109.2	C25—C24—H24B	109.5
C8—C9—H9B	109.2	C23—C24—H24B	109.5
H9A—C9—H9B	107.9	H24A—C24—H24B	108.1
C11—C10—C9	111.14 (15)	C26—C25—C24	111.55 (16)
C11—C10—H10A	109.4	C26—C25—H25A	109.3
C9—C10—H10A	109.4	C24—C25—H25A	109.3
C11—C10—H10B	109.4	C26—C25—H25B	109.3
C9—C10—H10B	109.4	C24—C25—H25B	109.3
H10A—C10—H10B	108.0	H25A—C25—H25B	108.0
C10—C11—C12	111.37 (15)	C25—C26—C21	110.54 (15)
C10—C11—H11A	109.4	C25—C26—H26A	109.5
C12—C11—H11A	109.4	C21—C26—H26A	109.5
C10—C11—H11B	109.4	C25—C26—H26B	109.5
C12—C11—H11B	109.4	C21—C26—H26B	109.5
H11A—C11—H11B	108.0	H26A—C26—H26B	108.1
			
O1—P1—N1—C13	−39.88 (14)	C8—C9—C10—C11	53.0 (2)
N3—P1—N1—C13	−165.92 (12)	C9—C10—C11—C12	−55.6 (2)
N2—P1—N1—C13	82.56 (14)	C10—C11—C12—C7	58.17 (19)
O1—P1—N2—C7	−172.79 (12)	N2—C7—C12—C11	178.05 (14)
N3—P1—N2—C7	−45.84 (14)	C8—C7—C12—C11	−58.12 (18)
N1—P1—N2—C7	65.08 (15)	P1—N1—C13—C18	160.06 (12)
O1—P1—N3—C1	75.27 (15)	P1—N1—C13—C14	−75.72 (16)
N2—P1—N3—C1	−44.69 (14)	N1—C13—C14—C15	−178.14 (13)
N1—P1—N3—C1	−166.34 (13)	C18—C13—C14—C15	−54.60 (19)
P1—N3—C1—C6	148.80 (13)	C13—C14—C15—C16	54.91 (19)
P1—N3—C1—C2	−86.58 (17)	C14—C15—C16—C17	−55.3 (2)
N3—C1—C2—C3	177.23 (14)	C15—C16—C17—C18	55.9 (2)
C6—C1—C2—C3	−57.97 (18)	N1—C13—C18—C17	−178.57 (14)
C1—C2—C3—C4	55.5 (2)	C14—C13—C18—C17	55.56 (19)
C2—C3—C4—C5	−53.4 (2)	C16—C17—C18—C13	−56.7 (2)
C3—C4—C5—C6	53.4 (2)	N4—C21—C22—C23	−177.38 (14)
N3—C1—C6—C5	−174.36 (14)	C26—C21—C22—C23	−55.43 (19)
C2—C1—C6—C5	58.95 (19)	C21—C22—C23—C24	54.4 (2)
C4—C5—C6—C1	−56.6 (2)	C22—C23—C24—C25	−54.3 (2)
P1—N2—C7—C8	152.71 (12)	C23—C24—C25—C26	55.5 (2)
P1—N2—C7—C12	−82.85 (17)	C24—C25—C26—C21	−56.7 (2)
N2—C7—C8—C9	−177.01 (14)	N4—C21—C26—C25	179.20 (14)
C12—C7—C8—C9	56.22 (19)	C22—C21—C26—C25	56.6 (2)
C7—C8—C9—C10	−53.8 (2)		

### Hydrogen-bond geometry (Å, º)

**Table:**

*D*—H···*A*	*D*—H	H···*A*	*D*···*A*	*D*—H···*A*
N2—H2*N*···O1^i^	0.87 (1)	2.14 (2)	3.0049 (18)	171 (2)
N3—H3*N*···O2^ii^	0.84 (1)	2.05 (2)	2.8837 (18)	173 (2)
N1—H1*N*···O3	0.86 (1)	2.21 (2)	3.0394 (18)	163 (2)
N4—H4*NC*···O1^iii^	0.89 (1)	2.05 (2)	2.9445 (18)	178 (2)
N4—H4*NB*···O3^iv^	0.89 (2)	1.94 (2)	2.7666 (19)	155 (2)
N4—H4*NA*···O2^v^	0.88 (2)	1.83 (2)	2.6992 (19)	169 (2)

Symmetry codes: (i) −*x*, −*y*, −*z*; (ii) −*x*, *y*−1/2, −*z*+1/2; (iii) *x*+1, *y*+1, *z*; (iv) −*x*+1, *y*+1/2, −*z*+1/2; (v) *x*+1, *y*, *z*.
